# Optimization-based decoding of Imaging Spatial Transcriptomics data

**DOI:** 10.1093/bioinformatics/btad362

**Published:** 2023-06-02

**Authors:** John P Bryan, Loïc Binan, Cai McCann, Yonina C Eldar, Samouil L Farhi, Brian Cleary

**Affiliations:** Klarman Cell Observatory, Broad Institute of MIT and Harvard, 415 Main St, Cambridge, MA 02142, USA; Department of Electrical Engineering and Computer Science, Massachusetts Institute of Technology, Cambridge, MA 02139, USA; Klarman Cell Observatory, Broad Institute of MIT and Harvard, 415 Main St, Cambridge, MA 02142, USA; Klarman Cell Observatory, Broad Institute of MIT and Harvard, 415 Main St, Cambridge, MA 02142, USA; Department of Electrical Engineering and Computer Science, Massachusetts Institute of Technology, Cambridge, MA 02139, USA; Department of Computer Science and Applied Mathematics, Weizmann Institute of Science, 218 Ullman, Rehovot 7610001, Israel; Klarman Cell Observatory, Broad Institute of MIT and Harvard, 415 Main St, Cambridge, MA 02142, USA; Program in Bioinformatics, Departments of Biomedical Engineering and Biology, Faculty of Computing and Data Sciences, Boston University, 665 Commonwealth Ave., Boston, MA 02215, USA

## Abstract

**Motivation:**

Imaging Spatial Transcriptomics techniques characterize gene expression in cells in their native context by imaging barcoded probes for mRNA with single molecule resolution. However, the need to acquire many rounds of high-magnification imaging data limits the throughput and impact of existing methods.

**Results:**

We describe the Joint Sparse method for Imaging Transcriptomics, an algorithm for decoding lower magnification Imaging Spatial Transcriptomics data than that used in standard experimental workflows. Joint Sparse method for Imaging Transcriptomics incorporates codebook knowledge and sparsity assumptions into an optimization problem, which is less reliant on well separated optical signals than current pipelines. Using experimental data obtained by performing Multiplexed Error-Robust Fluorescence *in situ* Hybridization on tissue from mouse brain, we demonstrate that Joint Sparse method for Imaging Transcriptomics enables improved throughput and recovery performance over standard decoding methods.

**Availability and implementation:**

Software implementation of JSIT, together with example files, is available at https://github.com/jpbryan13/JSIT.

## 1 Introduction

Imaging Spatial Transcriptomics (iST) methods, such as MERFISH (Chen *et al.* 2015), CosMx (He *et al.* 2022), and STARmap (Wang *et al.* 2018) simultaneously measure expression of targeted sets of hundreds of genes at a time with single molecule spatial resolution, revealing spatial patterns of cell-type arrangements and tissue organization. These newly developed methods have already allowed researchers to construct high-resolution spatial tissue atlases (Zhang *et al.* 2021), study subcellular compartmentalization of gene expression (Xia *et al.* 2019), and observe spatial differences in gene expression between phenotypical conditions (Moffitt *et al.* 2018), with potential for further discovery as datasets grow and analysis frameworks mature.

Highly multiplexed iST methods achieve gene multiplexing using combinatorial barcoding, in which each gene is assigned a distinct binary barcode from a pre-defined codebook (Tian *et al.* 2022). Fluorescent probes complementary to the genes are then iteratively applied, imaged at high resolution, and removed from the sample, such that an individual mRNA molecule (or transcript) only appears as a bright spot in the images corresponding to the ones in its barcode. Software pipelines then perform computational decoding of the acquired images, identifying the location of each fluorescent spot and attempting to assign it to a gene identity. These decoded transcripts are then assigned to a cell, and all transcripts within a cell are tallied up to produce a count table relating cell location to gene expression.

Notably, while single molecule resolution imaging is necessary to identify transcripts, once the count table is produced, most downstream methods do not need individual molecule locations to perform common tasks, such as cell-type identification and differential gene-expression analysis (Dries *et al.* 2021, Hu *et al.* 2021, Stogsdill *et al.* 2022). These analyses stand to be better empowered by profiling larger sample areas to increase the likelihood of capturing rare cell types and interactions and of profiling distinct regions of the tissue. However, the requirement of single molecule resolution imaging has thus far set the maximum imaging throughput to roughly 1 cm^2^ per day, limiting the biological discovery impact of iST. If, instead, imaging could be performed at lower magnification, larger amounts of tissue could be studied and iST could better enable the study of developmental time courses, comparisons among large numbers of patient samples, and the creation of large-scale tissue atlases.

In this work, we re-frame the decoding problem as an optimization problem and leverage algorithmic techniques like those used in super-resolution microscopy (Solomon *et al.* 2018), to enable decoding of lower magnification iST data. iST data are known to be structured: fluorescence signals are sparse in spatial coordinates. Currently used decoding pipelines, such as MERlin (Emanuel *et al.* 2020) (https://github.com/emanuega/MERlin), deconvolve optical signals and assign them to most likely barcodes in distinct steps, without taking full advantage of the known sparsity of the data. We instead present the Joint Sparse method for Imaging Transcriptomics (JSIT), which combines optical deconvolution and decoding of iST data, explicitly incorporating signal sparsity knowledge into the decoding step. In the process, this joint approach relaxes the requirement for high-magnification imaging, and increases overall throughput.

In the remainder of the article, we first provide a detailed description of the JSIT problem formulation and algorithm; its solution with the Fast Iterative Shrinkage-Thresholding Algorithm (FISTA), an iterative proximal gradient algorithm (Beck and Teboulle 2009, Eldar and Kutyniok 2012); and the metrics chosen to compare JSIT to MERlin. We then describe JSIT’s performance on real MERFISH data from the mouse somatosensory cortex (SSp) and primary motor cortex (MOp) imaged at both 40× and 60×, focusing specifically on JSIT’s ability to perform cell typing on low-resolution imaging data. We show that using JSIT on 40× data recapitulates the cell typing obtained by using MERlin on 60× data, while MERlin is not able to achieve this at 40×. We benchmark JSIT’s performance with several other decoding pipelines, including an optimization-based approach referred to as BarDensr (Chen *et al.* 2021), and the BlobDetector and MaxPeakFind pipelines from the Starfish software package (Perkel 2019); and further show that JSIT can accurately decode data acquired by other iST technologies. Finally, we discuss the practical throughput advantages of using JSIT to decode MERFISH data.

## 2 Materials and methods

### 2.1 Problem formulation: modeling iST data

**Figure 1:**
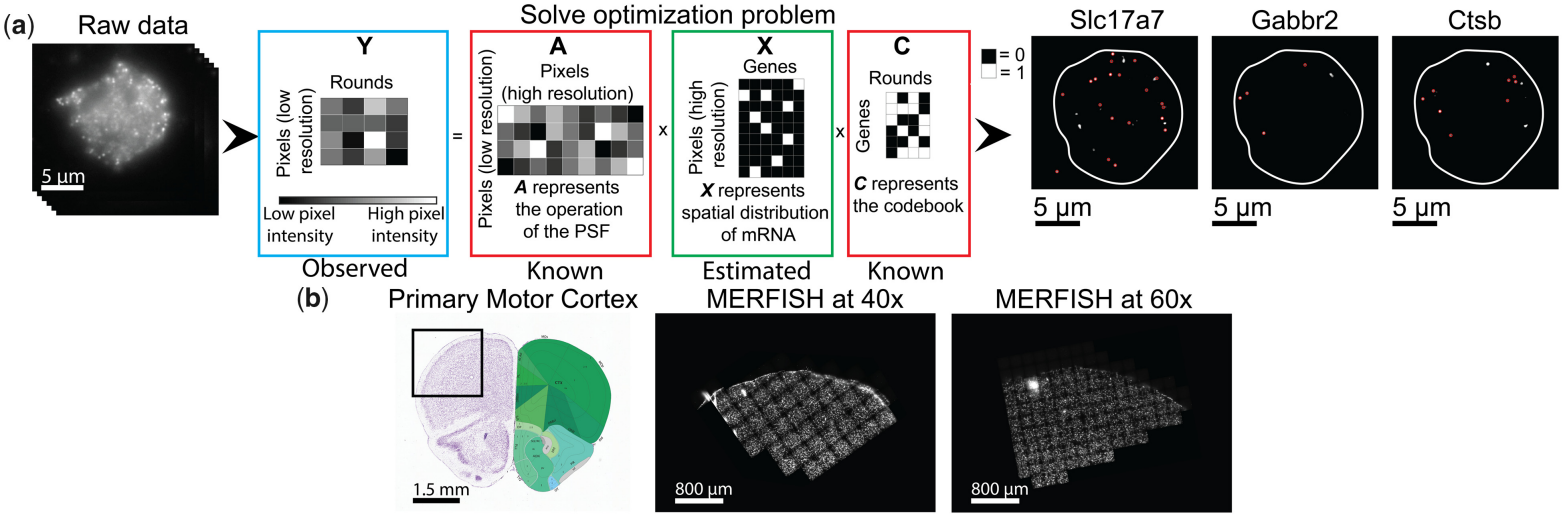
Summary of JSIT (a) schematic of JSIT pipeline. Left: MERFISH signal is acquired through multiple rounds of imaging. Center: MERFISH data are modeled as a matrix product. With this framing, decoding is a sparse recovery problem. Right: JSIT produces maps of the distribution of mRNA transcripts of each gene. (b) Schematic of experiment. Left: Allen Brain Atlas depiction of the mouse brain, with cell regions labeled on right. MOp highlighted (Lein *et al.* 2007). Center: downsampled MERFISH image of MOp, imaged at 40×. Right: downsampled MERFISH image of MOp, imaged at 60×.

We begin by modeling the iST data generation process, as depicted schematically in Fig. 1a. Our aim is to recover the spatial distribution and gene identity of mRNA from raw data consisting of F images with size n × n pixels, which encode gene identities with defined barcodes of F bits. We first vectorize and subsequently concatenate these images to form a matrix Y ∈ R^*N*_l_×F^, where $N_{l}$ = $n^{2}$ is the number of pixels in the field of view (FOV). Element $\left(i,f\right)$ of Y represents the pixel value at location i in the f-th image. We model the generation of Y as:


(1) Y=AXC+η.

Here, A represents the operation of the point-spread function (PSF) of the microscope, X represents the spatial distribution and gene identity of mRNA, which we aim to recover, C is the codebook, and η denotes additive experimental noise. This noise is not defined in specific terms, but is known to include low-frequency autofluorescence background, which varies strongly with location within the FOV, and high-frequency, uncorrelated shot noise.

To better resolve spots in the acquired data, we define the matrix X ∈ R*^N_h_×G^* at a higher resolution than Y, i.e. $N_{h}$>$N_{l}$. Specifically, we pick a scale factor s, and divide each pixel represented in Y into $s^{2}\;$equally sized sub-pixels, giving $N_{h}=s^{2}n^{2}=s^{2}N_{l}$. This scale factor can be chosen by the user: a higher scale factor will incur greater computational costs, but may provide more accurate results (Supplementary Fig. S1). Throughout the rest of the article, we use s=3. The non-zero values of elements of X indicate the intensity of fluorophores bound to mRNA molecules, while zeros indicate no mRNA present. Next, we define the PSF matrix A ∈ R^*N_l_*^^*×N_h_*^, such that element $\left(i_{l},i_{h}\right)$ represents the percentage of photons originating at location $i_{h}\;$in the sample distributed to pixel $i_{l}$ in the image; A can be obtained theoretically or empirically for any optical system used to image the sample. Finally, we define the codebook C by setting element $\left(g,f\right)$ of C ∈ Z^G×F^ equal to the value of bit f of barcode g. For any iST experimental protocol, C is known. Our goal is to recover X from Y.

### 2.2 Joint Sparse Method for Imaging Transcriptomics

#### 2.2.1 Preliminary denoising

To minimize the contribution of the noise term η, we filter both high- and low-frequency noise by convolving each of the F observed images with a difference-of-Gaussians band-pass filter, where one Gaussian is empirically chosen to be broader than the microscope PSF and the other narrower. This band-pass approach mitigates the effects of both high-frequency shot noise and low-frequency autofluorescence noise. In processing the 60× MERFISH data, e.g. we used the filter: where $r=\sqrt{x^{2}+y^{2}}$, with x and y representing distance in pixels. The resulting images are vectorized and concatenated to form $Y_{f}\;\in R^{N_{l}\times F}$.


(2) $$h\left(r\right)=\frac{1}{0.75\sqrt{2\pi }}e^{-\frac{1}{2}\frac{r^{2}}{0.75^{2}}}-\frac{1}{3\sqrt{2\pi }}e^{-\frac{1}{2}\frac{r^{2}}{3^{2}}},$$

#### 2.2.2 Decoding as a sparse recovery problem

We seek to recover X from $Y_{f}$ by solving a regularized least-squares optimization problem, using principles of compressed sensing (Eldar and Kutyniok 2012, Eldar 2015). We impose several constraints: **(**i) only one molecule is present at any given location, so the rows of X are one-sparse; **(**ii) molecules are present at few locations in the FOV, so X is row-sparse; **(**iii) the FOV contains few mRNA molecules, so X is overall sparse. We choose to impose constraint (i) in post-processing rather than as part of the optimization problem, finding that this improves results, similar to Mazor *et al.* (2018). We impose (ii) with a mixed $l_{1}/l_{2}$ norm on the rows of X, and (iii) by an $l_{1}$ norm on X. These functions are combined convexly, as in the Sparse Group LASSO formulation (Simon *et al.* 2013), leading to the optimization problem:


(3) $$\hat{X}={\text{argmin}}_{x}||Y_{f}-\mathrm{AXC}|{|}_{F}^{2}+\lambda_{1}\left(\left(1-\lambda_{2}\right)\Sigma_{i=1}^{N_{h}}\sqrt{\Sigma_{j=1}^{G}X_{i,j}^{2}}+\lambda_{2}||X|{|}_{1}\right).$$

We solve (3) with FISTA, using the proximal operator for the regularization function as given in Bach *et al.* (2012). These are both detailed in Supplementary Section S1.

Algorithm 1 JSIT

**Input:**Y, A, C, λ_1_, λ_2_, band-pass filter h, min. cluster size c, threshold $t_{x}$ on $\hat{X}$, max. FISTA iterations $i_{\text{max}}$

**Output:**$\hat{X}$

1: $\hat{X}$= $0_{N_{h},f}$

2: for columns $Y_{i}$ of Y**do**

3:    Y_f, i,:_ = vec(mat(Y_i_) **×**h)

4: **end for**

5: X^(1)^ ← FISTA(Y_f_, A, C, λ_1_, λ_2_, i_max_) [Supplementary Algorithm S1, solution to Equation (3) in Section 2.2.2]

6: Keep only maximum-value element of each row of X^(1)^

7: **for** elements ${\hat{X}}_{i,j}^{\left(1\right)}$of $\hat{X}$^(1)^ do

8:    **if**X^(1)^ < t_x_**then**

9:          ${\hat{X}}_{i,j}^{\left(1\right)}=0$

10:    **end if**

11: **end for**

12: **for** columns $\hat{X}$_i_ of $\hat{X}$_(1)_**do**

13:    B = bwconncomp(mat(${\hat{X}}_{i}$))

14:    **for** clusters B_j_ in B**do**

15:       if size(B_j_) ≥ c**then**

16:          $\mu_{j}$= round(centroid(B_j_))

17:          ${\hat{X}}_{\mu_{j},i}$= 1

18:       **end if**

19:    **end for**

20: **end for**

After obtaining $\hat{X}$, one-sparsity is imposed on the rows of $\hat{X}$ by setting to 0 all but the maximal value of each row of $\hat{X}$, rejecting small elements representing spuriously detected molecules. The resulting $\hat{X}$ is additionally hard-thresholded, to mitigate effects of low-intensity noise, and remove spots which poorly match the codebook. Having recovered $\hat{X}$, each column i of $\hat{X}$ is reshaped as an image, giving the spatial distribution of gene i. Clusters of non-zero elements are formed by identifying connected components using the Matlab command bwconncomp and the centroid of each cluster is taken to be the location of a molecule associated with gene i. This addresses cases where each molecule is represented by multiple non-zero elements in $\hat{X}$, due to small variations in the spot size, as can be seen in Fig. 1a. We reject clusters of pixels below an empirically chosen area threshold, to minimize spurious calls (typically 2).

The resulting method, called the JSIT, is summarized in Algorithm 1. For comparison, the MERlin decoding procedure is detailed in Supplementary Section S2.

#### 2.2.3 Post-processing and data cleanup

After the main decoding step, false**-**positive detections (possibly caused by artifacts of the hybridization chemistry, autofluorescence noise that escaped filtering, or low-intensity shot noise blurred together by the band-pass filter) are minimized by adaptively filtering decoded spots. We use a method very similar to MERlin (Moffitt *et al.* 2016), described in Supplementary Section S3. Decoded transcripts are then assigned to individual cells segmented using a seeded watershed approach (Meyer 1994); we reject cells with area below one-half of the median area or above two times the median area. Transcripts that fall within the boundaries generated by the segmentation are then assigned to cells, to construct a count table S**∈**R^*N_c_*^^×G^, where $N_{c}$ is the number of cells in the ROI, and element S_*i, j*_ is the number of decoded transcripts of gene j within the bounds of cell i. The centroids of the segmented areas of each cell are also obtained, and a table of locations is created, L**∈**R^*N_c_*^^×3^, with the i*-*th row of L a vector giving the 3D position in the ROI of the i-th cell. We also produce a table of “relative” locations, $L\left(r\right)$**∈**R^*N_c_*^^×3^, in which the i*-*th row is a vector giving the position within its FOV of the i**-**th cell.

Finally, we correct for observed expression differences between cells at the center and edges of the FOV, presumably caused by spherical aberrations of the microscope objective affecting imaging and decoding performance. We bin cells in all FOVs based on distance to the FOV center into K bins, and calculate the average expression level m_*g,k*_ for each of G genes, and scale the abundance of each gene in each cell by multiplying each element of S by s_*g,k*_ = m_*g,k*_**_,_**according to the bin k to which its cell belongs.

#### 2.2.4 Parameter tuning

Three parameters require tuning in the JSIT workflow: the regularization parameter λ_*1*_, the threshold for X, t_x_, and the minimum accepted cluster size t_c_ (λ_*2*_ can also be tuned, but here is set to 0.5 throughout). The parameter λ_*1*_ controls the sparsity level of the results: as λ_*1*_ is increased, more values of X are equal to zero, and fewer spots are decoded. However, the adaptive**-**filtering step complicates this: as λ_*1*_ is decreased, the number of spots decoded increases, and, along with this, the number of spots corresponding to blank barcodes increases. This can result in an increased number of coding barcodes being rejected, and a decrease in the total number of post**-**adaptive-filtering decoded spots, even as the total number of pre-adaptive-filtering decoded spots increases. We found that the best spatial homogeneity results are obtained when the number of post-adaptive-filtering decoded spots is maximal (see Section 2.3.2), and so we use this as a heuristic for the selection of λ_*1*_. To select λ_*1*_, we decode a small number of FOVs with various values of λ_*1*_, perform adaptive**-**filtering, and select the value which gives the highest number of post-filtering spots (Supplementary Fig. S2). After selecting λ_*1*_, we use the same method to select t_x_.

We also find that performance of the pipeline improves when clusters below a certain size are rejected before adaptive**-**filtering. Precision (% of detected molecules corresponding to real molecules) increases with the minimum cluster size c, as many small clusters represent noise, while recall (% of molecules detected) decreases as the minimal cluster size increases, because some real molecules are represented by small spots. We typically set c to 2.

### 2.3 Validation of JSIT

#### 2.3.1 Datasets

Prior to collecting experimental data, we performed preliminary simulations (Bryan *et al.* 2021), which suggested that 40× magnification is a reasonable compromise between quality and throughput: at lower magnification, blurring and loss of signal caused an increase in the false-positive rate in comparison to simulations with 40× data. We thus collected two experimental MERFISH datasets to compare the performance of JSIT and MERlin at 60× and 40× magnification. First, we obtained a small sample of mouse SSp, and prepared it for MERFISH imaging according to the procedure described in Moffitt *et al.* (2016). We probed the sample with a library of 20 genes, initially designed for sub-classification of microglia (Favuzzi *et al.* 2021). Readout and encoding probes were obtained from Vizgen Inc., and imaged using Vizgen’s MERSCOPE alpha instrument. The sample was imaged with both a 60× NA 1.4 objective, and a 40× NA 0.95 objective. We captured 31 *x*–*y* locations at 60× (20 *x*–*y* locations at 40×), and roughly 2000 cells. We acquired 7 *z*-positions per *x*–*y* location, separated by 1.5 µm.

We sought to image a larger sample, probed with a larger library of genes, with both 60× and 40× objectives, but it proved impractical to manually switch objectives at each FOV and still maintain high data quality at this larger scale (in particular, this resulted in issues of focal plane alignment across rounds). Instead, we opted to compare sequential tissue slices imaged at different magnification levels. We obtained two sequential coronal slices of mouse MOp, separated by 10 µm (Fig. 1b), and probed with a library of 115 genes, designed for cell typing in mouse cortex (Stogsdill *et al.* 2022). While this library was specifically designed to study the SSp, the SSp has similar cell-type structure to the MOp, and the library includes marker genes for the major cell types present in the MOp. One slice was imaged using the 60× NA 1.4 objective, and the other was imaged using the 40× NA 0.95 objective. At 60×, we captured 168 *x*–*y* locations covering 6.72 mm^2^ and roughly 6000 cells, and at 40× captured 51 *x*–*y* locations covering 4.59 mm^2^ and roughly 4500 cells. In both datasets, we acquired seven *z*-positions per *x*–*y* location, separated by 1.5 µm. Illumination intensities and exposure times were kept the same in each dataset (a longer exposure time could improve the signal-to-noise ratio in the 40× data, but would reduce the throughput gains for low-magnification imaging).

In addition to these datasets, we acquired to evaluate the performance of JSIT on low-magnification data, we tested JSIT on three publicly available iST datasets: a higher-plex MERFISH dataset (acquired at 60× magnification), studying the expression of 307 genes in mouse liver (Liu *et al.* 2023), a dataset using the *in situ* sequencing-based iST technology BARseq2 to study 67 genes in mouse cortex (Sun *et al.* 2021), and a small, single-FOV dataset acquired by the iST technology STARmap studying 160 genes (Wang *et al.* 2018).

#### 2.3.2 Cell cluster and localization analysis

After producing the cell-by-gene count table S, statistical results were computed on an aggregate level, calculating the average number of transcripts decoded per cell, the average number of genes with non-zero numbers of transcripts per cell, and the average intensity of a detected transcript (throughout the text, these results are expressed as a mean ± standard deviation unless otherwise noted. Statistical tests and *P***-**values are described next to individual results, with results treated as significant if *P* < .05, unless otherwise stated). Then, in the MOp data, standard single-cell analysis techniques were used to cluster cells by their gene**-**expression levels. All analysis was performed using the Scanpy library in Python (Wolf *et al.* 2018). The count table was first filtered to remove cells with zero counts, and genes which appeared in fewer than three cells. The count table was then normalized such that each cell would have the same number of total counts, and dimensionality reduction was performed using principal component (PC) analysis, keeping the top 50 PCs. We clustered the cells in PC space using the Louvain clustering algorithm (Blondel *et al.* 2008), selecting a resolution parameter by empirically adjusting until the major cortical cell types were revealed: excitatory neurons, interneurons, microglia, oligodendrocytes, and astrocytes. To maintain consistency in our evaluations of the 40× and 60× data, as well as in comparing the results of different decoding pipelines, we used the same Scanpy parameters for all results, including the Louvain resolution parameter (which we set to 0.65). Following this analysis, we removed *Slc17a7*, which was highly expressed in all excitatory neurons and repeated these steps to subcluster excitatory neurons to reveal cortical layer**-**specific subpopulations. We identified spatial regions belonging to major layers of the MOp—Layers 2/3, 4, 5, and 6—by visualizing the spatial expression of well-known marker genes of each layer (*Stard8*, *Rorb*, *Deptor*, and *Tle4*), and manually annotating the areas of high expression. To compare the cluster assignments in gene-expression space to the spatial layer assignments, we used the cluster homogeneity score (Rosenberg and Hirschberg 2007), defined as: where K is the number of spatial layers, C is the number of gene-expression space clusters, and $n_{c,k}$ is the number of cells belonging to cluster c and layer k.


(4) $$h=1-\frac{-\sum_{k=1}^{K}\sum_{c=1}^{C}\frac{n_{c,k}}{N}l\mathrm{og}\left(\frac{n_{c,k}}{\sum_{c=1}^{C}n_{c,k}}\right)}{\sum_{c=1}^{C}\frac{\sum_{k=1}^{K}n_{c,k}}{C}l\mathrm{og}\left(\frac{\sum_{k=1}^{K}n_{c,k}}{C}\right)},$$

This metric quantifies the notion that excitatory neurons assigned to the same cluster in gene-expression space should belong to the same spatial layer. This metric can obtain a high score even if a layer is comprised of multiple gene-expression space clusters, each confined to that layer. This is biologically reasonable as layers may be subdivided into multiple cell types. The homogeneity score is related to the ratio of the conditional entropy of layers given cluster assignments to the overall entropy of the layers. The score is between 0 and 1, with 1 signifying perfect homogeneity.

## 3 Results

### 3.1 JSIT accurately decodes high-magnification data

We benchmarked the performance of the JSIT and MERlin pipelines on high- and low-magnification MERFISH data by analyzing two datasets, which we acquired at 60× and 40× magnifications. We processed a small sample of mouse SSp, with a 20-gene MERFISH library, in which the same tissue slice was imaged with both 60× and 40× magnification lenses, and two sequential slices of mouse MOp, one acquired with 60× and the other with 40×, with a 115-gene MERFISH library.

**Figure 2:**
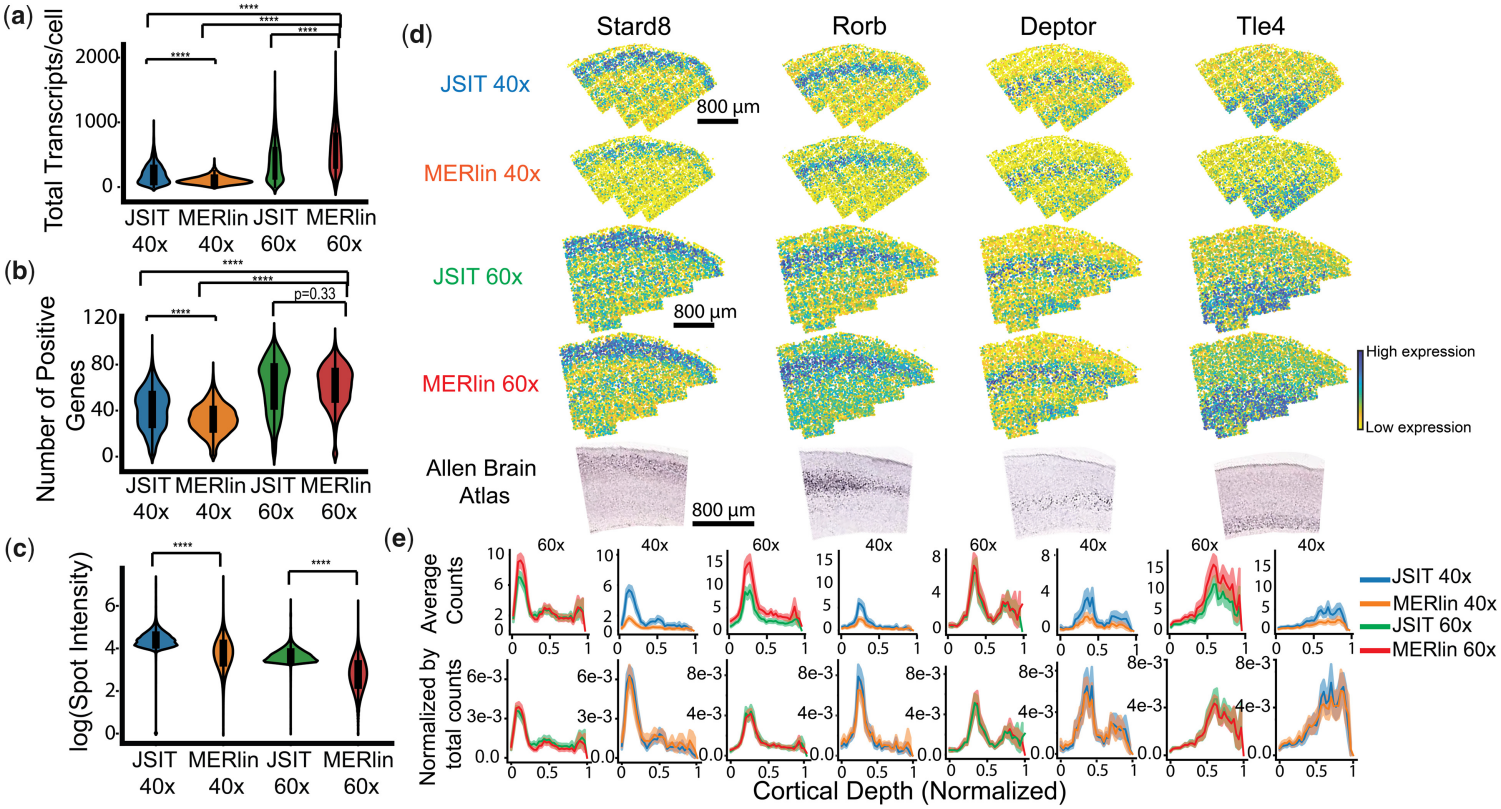
JSIT accurately recovers gene expression. (a–c) Transcript quantification in decoded results, by decoding pipeline. Asterisks indicate that differences between distributions are statistically significant. (a) Number of transcripts per cell. (b) Total genes with non-zero counts per cell. (c) Intensity of decoded molecules. (d) Spatial distribution of several genes with known spatial patterns as decoded by MERlin and JSIT from 40× and 60× data, and spatial distribution of genes as given by the ABA (Lein *et al.* 2007). (e) Spatial distribution of expression of spatially varying genes versus cortical depth. Top row: average number of mRNA molecules per cell, in each depth bin. Bottom row: average number of mRNA molecules per cell, normalized by total counts.

On aggregate measures of transcripts, there was good agreement between JSIT and MERlin results in the mouse brain datasets. We first measured pseudo-bulk gene abundances in both 60× datasets, and found high-gene-expression correlation between JSIT and MERlin (in the SSp data, Pearson’s r = 0.94, in the MOp data, Pearson’s r = 0.95). Log-transformed pseudo-bulk abundances also correlated strongly (SSp: Pearson’s r = 0.98, MOp: Pearson’s r = 0.83), as did gene expression in individual cells (SSp: Pearson’s r = 0.93, MOp: Pearson’s r = 0.91). MERlin detected a significantly larger number of transcripts per cell relative to JSIT in the MOp data (587 ± 342, *n *= 6068 cells versus 400 ± 291, *n *= 5809 cells, *P *= 6.7 × 10^−217^, Welch’s *t*-test), while in the SSp data, JSIT showed a modest but significant increase in the number of detected transcripts per cell (188 ± 156, *n *= 2064 cells versus 167 ± 143 *n *= 2064 cells, *P *= 7.1 × 10^−6^, Welch’s *t*-test). MERlin and JSIT did not detect a significantly different number of genes with non-zero counts per cell (SSp: 12 ± 5, *n *= 2064 cells versus 12 ± 4 *n *= 2064 cells, *P *= .25, Welch’s *t*-test. MOp: 61 ± 18, *n *= 6068 versus 60 ± 22, *n *= 5809, *P *= .33, Welch’s *t*-test) (Fig. 2a). Because ground truth data are difficult to acquire in parallel with MERFISH, it is not clear whether the additional calls by MERlin represent true or false positives. To understand the apparent higher sensitivity of MERlin in comparison to JSIT in the MOp, we thus calculated the average pixel intensity of the signal from decoded transcripts in the MOp data, finding that the average pixel intensity for MERlin calls was lower than that of JSIT calls (19 ± 15 post-denoising counts, *n *= 8.4 × 10^6^ versus 40 ± 16, *n *= 4.5 × 10^6^, *P* < 2.2 × 10^−308^, Welch’s *t*-test) (Fig. 2a). This was also apparent qualitatively: when examining the raw data for individual transcript calls, JSIT’s calls generally are brighter than MERlin’s (Supplementary Fig. S3). Examining the correlation of pseudo-bulked log-transformed gene expression showed that JSIT detected a consistently lower ratio of counts for each gene relative to MERlin (Supplementary Fig. S4). We broke down this analysis by cell type, investigating the predicted disjoint expression of a marker gene for inhibitory neurons (*Erbb4*) and a marker for microglia (*Tmem119*) in both cell types by both pipelines (Supplementary Fig. S5). We again found that MERlin detects higher counts of each gene in each cell type than JSIT.

We noted that the two mouse brain datasets were quite similar, and MERFISH data can have quite different characteristics in other tissues (e.g. cells may be more or less densely packed, or be differently shaped). Further, MERFISH data plex can be larger than the 20 and 115 gene panels, we worked with so far. Thus, we tested JSIT’s ability to decode publicly available data from a 307-gene MERFISH experiment studying mouse liver, imaged at 60×, and compared the results to the gene-expression results reported in the publication (Liu *et al.* 2023). The pseudo-bulk gene expression of the decoded results from JSIT were highly correlated with the results from the paper (Pearson’s r = 0.95), as were the log-transformed pseudo-bulk gene expression (Pearson’s r = 0.79, and gene expression in individual cells (median Pearson’s r = 0.73) (Supplementary Fig. S6). While good, these results were poorer than the MOp and SSp results—it is worth noting that these particular liver samples showed relatively poor correlation to pseudo-bulk RNA seq in the publication (Pearson’s r = 0.61). Thus, without ground truth it is hard to know whether lower correlation to MERlin necessarily means that JSIT is less successful with higher-plex codebooks.

**Figure 3:**
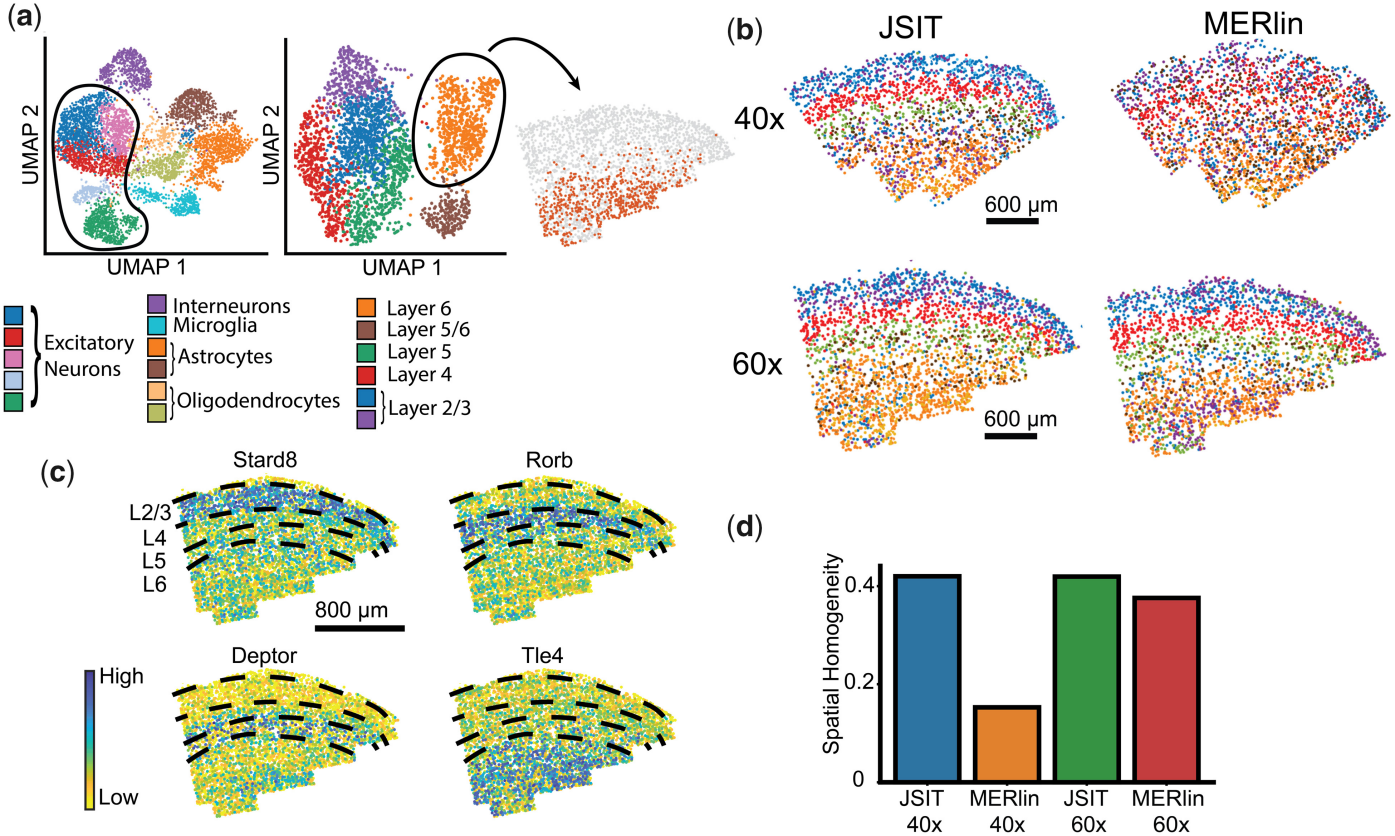
JSIT accurately recovers cortical layers of excitatory neurons (a) UMAP plots of the gene-expression cells decoded by JSIT, imaged at 60× magnification. Left: all cells, with clusters associated with excitatory neurons outlined. Center: re-clustering of excitatory neurons. Right: mapping of one cluster (L6 excitatory neurons) to spatial coordinates. (b) Spatial distribution of cell types identified by unsupervised clustering of excitatory neurons in results from JSIT and MERlin, on 40× and 60× data. (c) Defined spatial layers of excitatory neurons in the cortex (2/3, 4, 5, and 6), in 60× data, overlaid on distribution of key marker genes as detected in JSIT 60× data. (d) Spatial homogeneity is computed between cell-type clusters and spatial layers shown in (c).

Moving from aggregate to spatially resolved analysis, we examined the spatial distribution of the expression of several marker genes of cortical layers in the MOp (*Stard8*, *Rorb*, *Deptor*, and *Tle4*). We found that in both MERlin and JSIT, expression qualitatively matched those recorded in the Allen Brain Atlas (ABA) (Lein *et al.* 2007) (Fig. 2b). When we measured the distribution of these genes as a function of cortical depth, and normalized by the total number of transcripts detected, JSIT and MERlin produced nearly identical distributions, though MERlin showed higher transcript numbers overall (Fig. 2c). Next, we investigated JSIT and MERlin’s ability to produce results which would accurately identify cell type, comparing the results of clustering data processed by JSIT and MERlin by gene expression. It is well-known from scRNA-seq data that cortical excitatory neurons in different layers of the mouse brain have distinct expression profiles (Zhang *et al.* 2021). Thus, unsupervised clustering of neurons by gene expression should result in clusters corresponding to layer-specific subtypes. When we performed this analysis, we found that for both MERlin and JSIT, the cell-type clusters mapped to regions lying within the well-known cortical layer boundaries (Fig. 3a). We also computed spatial homogeneity, and found JSIT produced clusters with spatial homogeneity of 0.41, higher than the spatial homogeneity of 0.38 achieved by MERlin (Fig. 3d), showing that JSIT reproduces the laminar spatial structure of cortical neurons at least as well as MERlin. As a whole, these results showed that, with 60× high-magnification data, the results produced by JSIT were very similar to those produced by MERlin, with slightly lower sensitivity.

### 3.2 JSIT enables higher throughput by accurately decoding low-magnification MERFISH data

While our 60× analysis showed that JSIT was accurate, if less sensitive than MERlin, our goal was to apply it to lower magnification data to increase the throughput of the overall data generation. We thus performed the same set of analyses on the results produced by processing the SSp and MOp 40× data with MERlin and JSIT, starting with transcript-level aggregate metrics. We also processed the MOp 40× data with two decoding pipelines from Starfish (Perkel 2019), BlobDetector and MaxPeakFind, and compared their results to MERlin and JSIT to evaluate their accuracy in decoding low-magnification MERFISH data. Pseudo-bulk correlation with the MERlin 60× data was high for both JSIT 40× (in the paired SSp data, Pearson’s r = 0.98, in the unpaired MOp data, Pearson’s r = 0.94) and MERlin 40× (SSp: Pearson’s r = 0.92, MOp: Pearson’s r = 0.97), but much lower for the Starfish pipelines (MOp: Pearson’s r = 0.47 for BlobDetector, Pearson’s r = 0.68 for MaxPeakFind). When pseudo-bulk abundances were log-transformed, correlation remained high for JSIT (SSp: Pearson’s r = 0.84, MOp: Pearson’s r = 0.93), and MERlin (SSp: Pearson’s r = 0.92, MOp: Pearson’s r = 0.97), and was very low for the BlobDetector and MaxPeakFind (MOp: Pearson’s r = 0.16 and 0.19, respectively). We concluded that the Starfish pipelines were unsuitable for processing low-magnification data.

Comparing MERlin and JSIT further showed that both detected significantly fewer transcripts per cell at 40× than MERlin detected at 60× (SSp, MERlin 40×: 27 ± 38, *n *= 2064, JSIT 40×: 111 ± 127, *n *= 2064, MOp, MERlin 40×: 104 ± 60, *n* =  4323, JSIT 40×: 200 ± 144, *n *= 4288, Welch’s *t*-test gives *P* < 2.2 × 10^−308^ for both MERlin 40× and JSIT 40× in comparison to MERlin 60× in both datasets), but JSIT 40× detects significantly more transcripts per cell than MERlin 40× (SSp: *P *= 1.1 × 10^−163^, MOp: *P* < 2.3 × 10^−308^, Welch’s *t*-test). The same pattern is seen in computing mean genes with non-zero counts per cell, with both MERlin 40× and JSIT 40× detecting significantly fewer positive genes per cell than MERlin 60× (in SSp, MERlin 40×: 4 ± 3, JSIT 40×: 8 ± 5, in MOp, MERlin 40×: 32 ± 13, JSIT 40×: 41 ± 17, *P* < 2.2 × 10^−308^, Welch’s *t*-test for both in comparison to MERlin 60×). JSIT 40×, however, detected significantly more positive genes per cell than MERlin 40× (SSp: *P *= 8.8 × 10^−167^, MOp: *P *= 1.1 × 10^−139^, Welch’s *t*-test).

As in the 60× analysis, we found that the average decoded spot intensity was higher for JSIT than MERlin (87 ± 43 post-filtering counts, *n *= 1.6 × 10^6^ versus 53 ± 41, *n *= 1.2 × 10^6^, *P* < 2.2 × 10^−308^, Welch’s *t*-test) (Fig. 2a). On a gene-by-gene basis, JSIT 40× and MERlin 40×’s count numbers were smaller than MERlin 60×’s by a consistent ratio (Supplementary Fig. S4b and c), though JSIT 40×’s decrease is smaller than MERlin 40×’s. Surprisingly, we found a better correlation between (log-transformed) pseudo-bulked JSIT 40× compared to MERlin 60× than we did for JSIT 60× versus MERlin 60× (Pearson’s r = 0.93 versus 0.83). The difference in sensitivity between JSIT 40× and MERlin 40× remained when examining the disjoint expression of *Tmem119* and *Erbb4* in microglia and inhibitory neurons, with JSIT 40× finding significantly more counts of the positive marker genes than MERlin 40×, though not quite so many as were found in the 60× data (Supplementary Fig. S5). We thus conclude that when processing 40× data, JSIT and MERlin both provide accurate results, but JSIT is much more sensitive, with results close to the performance of both JSIT and MERlin at 60×.

When comparing spatial patterns of gene expression, the JSIT 40× results look qualitatively similar to the JSIT 60× results and the ABA, while the results of using MERlin 40× show sparser expression patterns, especially for *Stard8* (Fig. 2d). As a function of cortical depth, we observed the same pattern: for each gene, JSIT detected substantially more transcripts in the region for which the gene is a marker, and the normalized frequency distribution generated by the JSIT had a higher peak and lower trough than that generated by MERlin (Fig. 2e). Patterns of gene expression were much less well-defined when data were processed with the Starfish pipelines (Supplementary Fig. S7c). The BlobDetector results exhibited strong variation between neighboring FOVs, and the MaxPeakFind results had a high false-positive rate, with genes known to be spatially confined detected in large numbers outside the expected layer.

While noting that 40× data analyzed by JSIT had lower detection sensitivity than 60× data, the qualitative similarity between the MERlin 60× and JSIT 40× gene distributions led us to consider whether we could still use JSIT 40× data to perform the types of cell-based downstream analyses that iST typically targets. We thus clustered the cells of both the JSIT 40× and MERlin 40× count tables by gene expression and computed the spatial homogeneity of sub-clustered excitatory neurons. The gene-expression clusters from JSIT map well to the cortical layers, as defined by expression of marker genes (Fig. 3c). The gene-expression clusters from MERlin 40× are much less spatially confined (Fig. 3b). The JSIT 40× results have spatial homogeneity of 0.40, higher than even the MERlin 60× results (0.38), while the MERlin 40× results have much lower spatial homogeneity, 0.18 (Fig. 3d). We noted that spatial homogeneity varied slightly with the Louvain cluster parameter, but that different datasets’ relative performance on this metric did not change substantially as the Louvain parameter varied (Supplementary Fig. S8). Unsupervised clustering of the results from BlobDetector and MaxPeakFind gave poor results (Supplementary Fig. S7d), with spatial homogeneity of 0.05 for BlobDetector and 0.09 for MaxPeakFind. So, while JSIT may exhibit lower sensitivity when decoding 40× data, it is nonetheless able to produce expression clusters that match the laminar structure in this data as well as MERlin on 60× data, and much better than other pipelines on this data.

**Figure 4:**
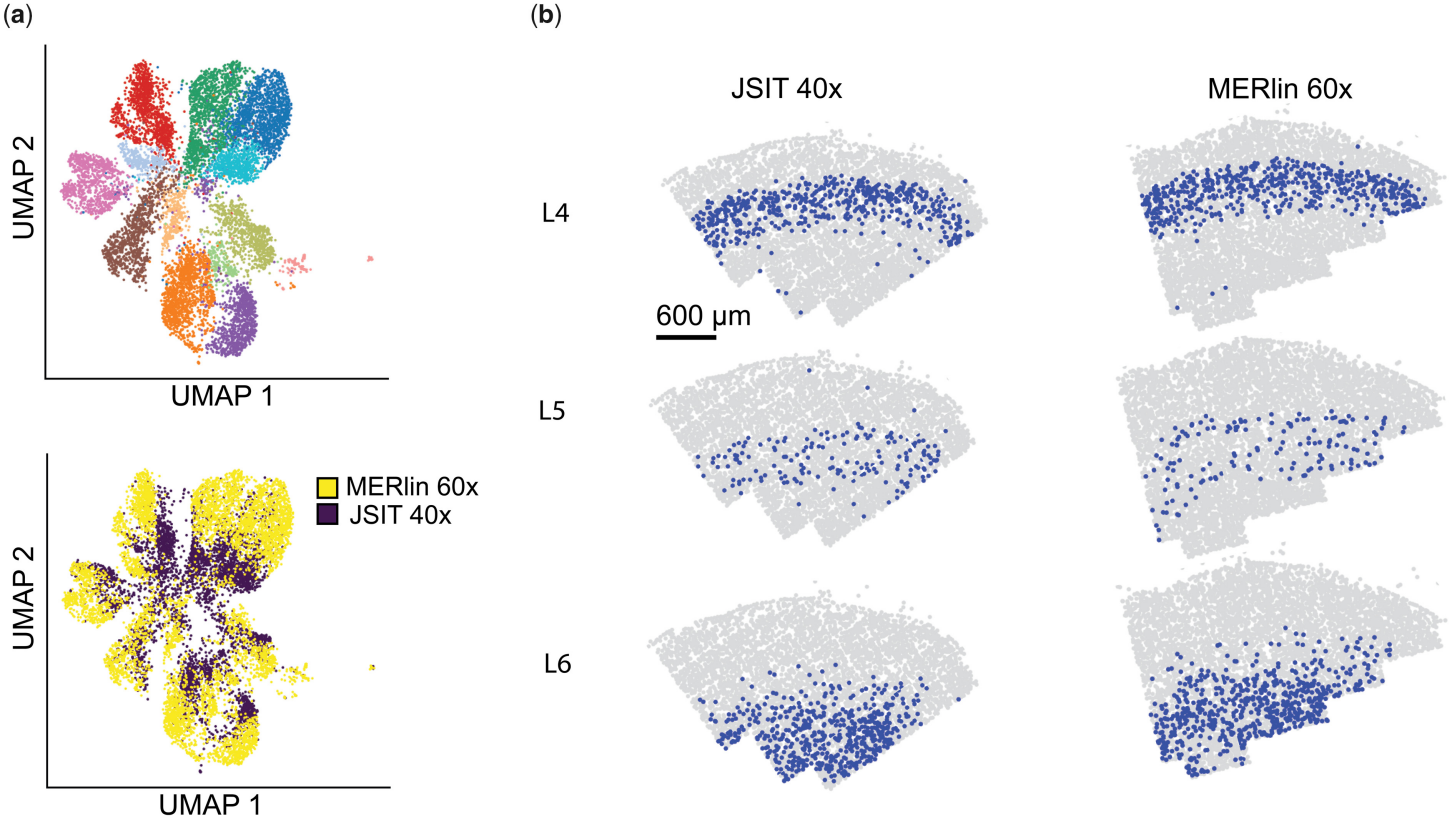
JSIT 40× clustered with MERlin 60× (a) UMAP, and clustering with Louvain clustering algorithm, of JSIT 40× results together with MERlin 60× results. (b) Spatial mapping of cells clustered as excitatory neurons, when JSIT 40× and MERlin 60× results are clustered together.

To test the similarity of the cell-type clusters found by JSIT 40× and MERlin 60×, we clustered the JSIT 40× and MERlin 60× results together. Despite some batch effects, we noted that all but two clusters (which correspond to the JSIT 40× and MERlin 60× clusters for L2/3 excitatory neurons) contained cells from both JSIT 40× and MERlin 60× (Fig. 4a), showing that we obtain the same major cell types in the results from both JSIT 40× and MERlin 60×. Additionally, we found that clusters labeled as L4, L5, and L6 excitatory neurons mapped to the same spatially confined layers in the JSIT 40× and MERlin 60× data (Fig. 4b). This confirmed JSIT’s ability to reproduce the clustering results of MERlin 60× at 40× magnification, enabling higher throughput. We also performed joint clustering with all four datasets (JSIT 60×, MERlin 60×, JSIT 40×, and MERlin 40×), and found that the pipelines’ results were well-integrated in many clusters, i.e. that several clusters contained cells from all four pipelines. In clusters corresponding to excitatory neurons, we found divergence between the two pipelines, with JSIT 40× and 60× clustering together and MERlin 40× and 60× clustering together (Supplementary Fig. S9a and b). We subclustered the excitatory neurons, again clustering the JSIT and MERlin results together, computed spatial homogeneity for the resulting neuronal subtypes. In this subclustering, we again saw that the JSIT results clustered together, and the MERlin results clustered together (Supplementary Fig. S9c and d). The MERlin 40× results benefited from integration into the neighborhood graph with the MERlin 60× results, increasing in spatial homogeneity in comparison to the results of clustering separately, although they remained substantially lower than the MERlin 60×, JSIT 60×, and JSIT 40× results (Supplementary Fig. S9d and e). This gives further evidence that, while JSIT and MERlin do not produce identical results, JSIT accurately identifies cell types, and does so more accurately at 40× than MERlin.

In all, we found that JSIT enables accurate cell typing of MERFISH when imaging at 40×, with somewhat lower sensitivity than when imaging at 60×. In the common scenario in which the biological question in centered on accurate cell typing, JSIT enables increased experimental throughput. Thus, JSIT allows the profiling of larger volumes of tissue and allow transcriptional study of broader spatial contexts, which is necessary to better understand larger-scale systems of genetic behavior.

### 3.3 JSIT decodes MERFISH data more accurately than BarDensr

In addition to MERlin, we compared JSIT to BarDensr (Chen *et al.* 2021), another optimization-based iST-decoding pipeline that was designed for use on the BARseq2 iST technology (Sun *et al.* 2021). To compare the performance of BarDensr to that of JSIT, we first compared the performance of JSIT and BarDensr in decoding simulated iST data (with known ground truth) available with the BarDensr software package. On this synthetic data, JSIT exhibited a significantly lower false-positive rate than BarDensr, and comparable true-positive rate (Supplementary Section S4). We saw a similar pattern when we compared the performance of JSIT and BarDensr in decoding experimental data. We processed four 1000×1000 pixel patches of the 60× MOp data with BarDensr, using the procedure provided in the example notebook provided in the BarDensr documentation, and compared the resulting gene expression to the results obtained by MERlin. Pseudo-bulk gene expression were highly correlated (Pearson’s r = 0.97, as were log-transformed pseudo-bulk gene expression (Pearson’s r = 0.81). In comparison, in the JSIT results for these four patches pseudo-bulk gene expression were equally highly correlated (Pearson’s r = 0.97, and log-transformed pseudo-bulk gene expression were more highly correlated than BarDensr (Pearson’s r = 0.91). In investigating these results, we hypothesize that BarDensr is sensitive to noise in MERFISH data, possibly because it was built for *in situ* sequencing methods, which produce much brighter signals, and have substantially less noise than MERFISH data. BarDensr infers a higher expression level than JSIT for genes, which were lowly expressed in the MERlin results (Supplementary Fig. S10a and b).

To further evaluate the accuracy of JSIT and BarDensr’s results, we compared the expression levels of known cell-type marker genes in the JSIT and BarDensr results (Supplementary Fig. S10c). This analysis showed that BarDensr is somewhat more sensitive than JSIT, detecting more counts of marker genes in cells, which belong to the corresponding cell type. However, BarDensr also calls many more counts of the marker genes in cells, which do not seem to belong to the corresponding cell type—a much higher apparent false-positive rate than JSIT. We conclude that JSIT more accurately decodes MERFISH data, in particular being less sensitive to noise, and thus more accurately inferring the expression of lower-expression genes.

### 3.4 JSIT accurately decodes data from other iST technologies

**Figure 5:**
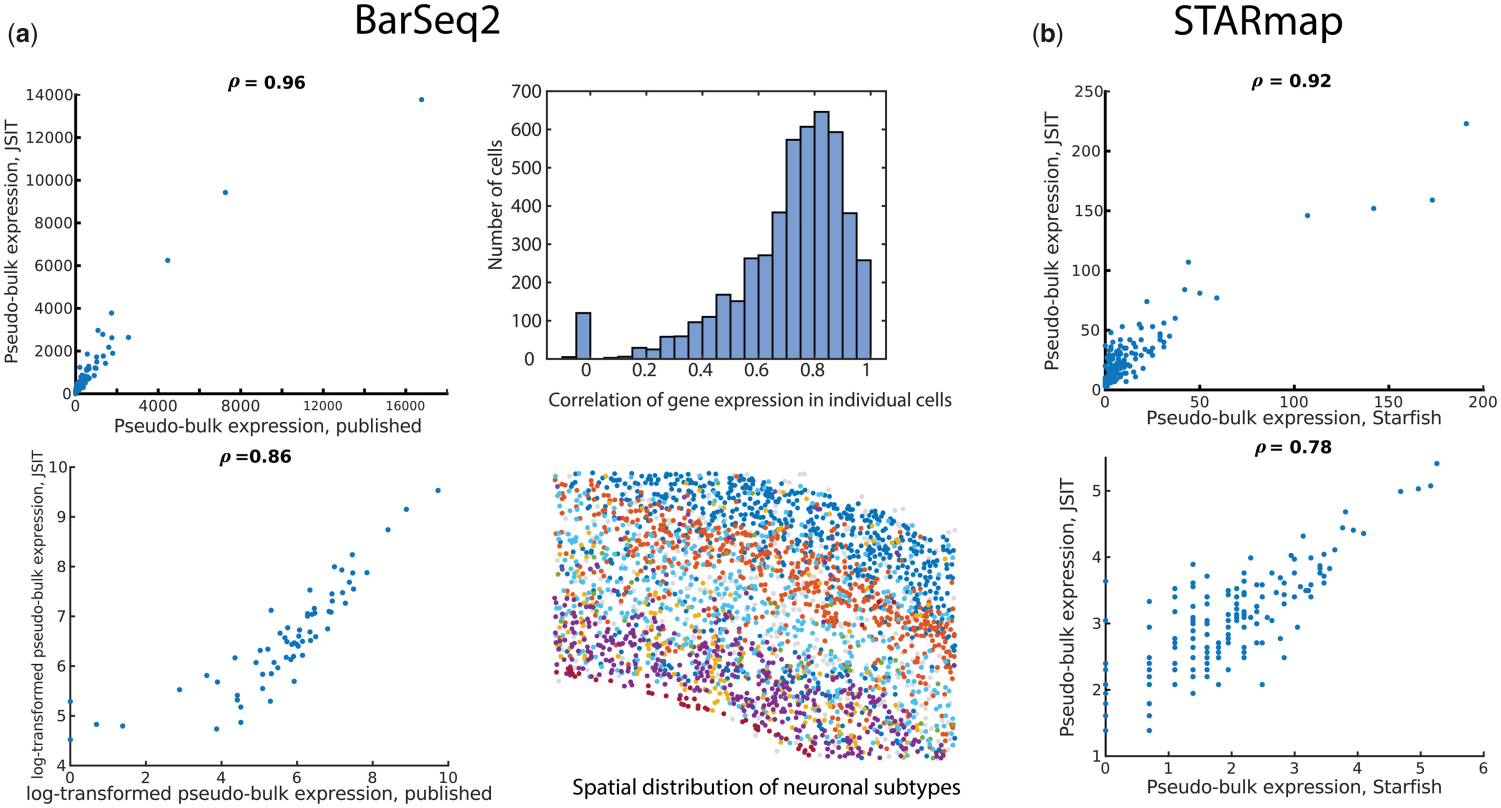
JSIT recapitulates published iST results (a) left: pseudo-bulk gene expression and log-transformed pseudo-bulk gene-expression comparison between JSIT and published data in 67-gene BARseq2 mouse cortex study. Right top: histogram of gene-expression correlation between JSIT and published data in individual cells. Right bottom: spatial distribution of neuronal subtypes obtained from JSIT results, revealing laminar structure. (b) Pseudo-bulk gene expression and log-transformed pseudo-bulk gene-expression comparison between JSIT and published data in 160-gene STARmap study.

Finally, we tested whether JSIT could successfully decode iST data produced using other protocols, which can produce differently structured data than MERFISH (e.g. different signal-to-noise ratios, or differently structured codebooks). We ran the JSIT pipeline on two publicly available datasets, acquired using two different iST technologies, BARseq2 and STARmap. As shown in Fig. 5, JSIT produced results matching the published results in both datasets. In each case, we computed pseudo-bulk gene expression, log-transformed pseudo-bulk gene expression, and the gene expression in each cell. In the BARseq2 dataset, pseudo-bulk gene expression was highly correlated (Pearson’s r = 0.97) as was log-transformed pseudo-bulk gene expression (Pearson’s r = 0.89) and gene expression in individual cells (median Pearson’s r = 0.76). Additionally, performing cell typing by gene expression on neurons with the JSIT results, according to the procedure detailed in Sun *et al.* (2021), produced laminar arrangements of neuronal cell types, which matched the arrangement in Fig. 3b of the BARseq2 publication (Fig. 5a). In the STARmap data, pseudo-bulk gene expression was highly correlated (Pearson’s r = 0.92), as was log-transformed pseudo-bulk gene expression (Pearson’s r = 0.78). Individual cells also had highly correlated gene expression (median Pearson’s r = 0.81) (Fig. 5b). We thus conclude that JSIT is a broadly useful decoding tool for multiple types of iST data.

## 4 Discussion

We have presented a new algorithmic pipeline, JSIT, for decoding multiplexed iST data. JSIT uses an optimization-based method to take advantage of the sparse nature of iST signals, and, with high-magnification imaging, shows comparable ability to detect gene expression, and perform cell typing, in comparison to the currently used decoding pipeline, MERlin.

We found that JSIT decoded MERFISH data acquired using a 40× microscope objective with higher sensitivity than MERlin on the same data, although lower sensitivity than MERlin’s decoding of 60× data. Despite this drop of sensitivity relative to 60× data, when the gene count matrix obtained by decoding 40× data with JSIT is clustered by gene expression using standard pipelines, clusters corresponding to all major cell types are correctly identified, with higher spatial homogeneity than that obtained by using MERlin to decode 60× data. In contrast, the gene count matrix obtained by decoding 40× data with MERlin was unable to produce cell-type clusters, which correctly correspond to cell types, especially in excitatory neurons. We conclude that decoding with JSIT enables accurate cell typing while imaging at lower magnification.

Performing a MERFISH experiment with 40×, rather than 60×, lens, will reduce imaging time substantially. In concrete terms, a six-round MERFISH experiment will save 1040 min (about 17 h) per cm^2^ of tissue imaged by imaging at the lower magnification level. Trade-offs, of course, exist: transcript detection sensitivity is somewhat lower in 40× data decoded by JSIT, in comparison to 60× data decoded by MERlin. Additionally, given the scale of iST data (frequently terabytes), it is important to consider the computational resources required to implement JSIT. Because the JSIT algorithm involves many large matrix multiplications, it requires substantially more computational time per FOV than MERlin: almost 57-fold more for the same area (Supplementary Fig. S11). While this is a significant difference, we note that MERlin performance has been optimized several times, while this is the first presentation of JSIT; that computational load is easier to parallelize than experimental load; and that computational costs for MERFISH experiments are dominated by storage rather than by compute. In our cost models, the decreased storage costs of lowering the number of FOVs stored by 2.25× pays for the difference in compute time expense in the first year of the project. So, in cases in which computational resources are readily available, and in which the key priority is identification of cell types, JSIT can be used to study larger tissue samples in less time, enabling a greater range of insights into relationships between different cell types. Additionally, if computational costs are of particular concern to the user, the scale factor s may be reduced: in this article, we have set s = 3 throughout, but setting s = 1 will reduce computational costs by 60%, with a small sacrifice in performance (Supplementary Fig. S1).

We note that, at both 40× and 60× magnification, JSIT is less sensitive than MERlin 60×. We hypothesize that a key factor limiting sensitivity is the adaptive-filtering step, which may inadvertently remove correctly identified spots along with spurious spots. In future work, two key avenues can be explored to remedy this. First, more spurious spots should be removed before performing adaptive-filtering, so that less aggressive filtering will be required, and more correctly identified spots will be retained. This could be done by improving the preliminary denoising steps, by, for instance, explicitly estimating and removing the high-frequency background shot noise, and more effectively removing low-frequency autofluorescence noise produced by out-of-focus signal by 3D deconvolution. Second, the adaptive-filtering process may be improved, so fewer correctly identified spots are filtered out. To accomplish this, additional metrics could be incorporated by which to filter out spurious spots, such as a morphological metric (spurious spots will be less likely to be round), and the ratio of the probability of the spot being called as the most likely barcode to the probability of assignment to the second-most likely barcode (spurious spots will have “messier” barcodes and this ratio is likely to be higher). Together, these two approaches of improved initial denoising and improved adaptive-filtering may improve sensitivity and increase accuracy.

While in this study, we have focused on the application of JSIT to MERFISH data, we have also shown that this optimization-based method may be applied to other multiplexed iST methods, such as BARseq2 (Sun *et al.* 2021), or STARmap (Wang *et al.* 2018). We suggest that JSIT or an adapted version be applied widely to other iST strategies. Finally, JSIT is readily extensible due to its straightforward model of iST signal generation: more complex noise models may be incorporated, e.g. or JSIT may be extended to a 3D analysis of IT data sampled more densely by depth. The optimization-based approach also opens the door to the incorporation of machine learning via algorithm unrolling (Monga *et al.* 2021, Sahel *et al.* 2022), as in Dardikman-Yoffe and Eldar (2020), which can also reduce computational costs. Extensions are likely to improve the performance of JSIT and improve ability to study the spatial distributions of gene expression. Therefore, JSIT helps lower experimental overhead by increasing iST throughput and offers a framework for a general iST-decoding tool.

## Supplementary Material

btad362_Supplementary_Data

## Data Availability

Software implementation of JSIT, together with example files, are available at https://github.com/jpbryan13/JSIT. Raw MERFISH data is available from the authors on request.
