# Supplementary material for: Optimization-based decoding of Imaging Spatial Transcriptomics data
Source: Bioinformatics. 2023 Jun 2;39(6):btad362. doi: 10.1093/bioinformatics/btad362 (PMC10287917; doi:10.1093/bioinformatics/btad362)
Supplement: btad362_Supplementary_Data [file btad362_supplementary_data.pdf]

Optimization-Based Decoding of Imaging Spatial Transcriptomics Data

Supplementary Material

Contents

S1 FISTA algorithm to solve Eqn. 3 (Section 2.3)

S2 Detailed MERlin decoding algorithm

S3 Adaptive filtering to remove false positives

S4 JSIT and BarDensr on simulated data

S5 Supplemental Figures

- **Figure S1** Compute-performance trade off with scale factor
- **Figure S2** Parameter selection of λ and t_x
- **Figure S3** Individual spot decoding from raw MERFISH data
- **Figure S4** Log-transformed pseudo-bulk expression by pipeline in MOp data
- **Figure S5** Abundance of marker genes in different cell types
- **Figure S6** JSIT decodes high-plex mouse liver MERFISH data
- **Figure S7** Performance of Starfish decoding pipelines
- **Figure S8** Spatial Homogeneity varies with Louvain resolution parameter
- **Figure S9** Clustering JSIT and MERlin datasets together
- **Figure S10** Comparison of JSIT and BarDensr

- **Figure S11** Compute time requirements of decoding pipelines

S1 FISTA for JSIT

Algorithm S1 FISTA solution of Eqn. 3 (Section 2.3)

Input: Y, A, C, λ_1, λ_2, i_{max}
Output: $\hat{X}^{(k)}$

$K = CC^T$
$M = AA^T$
$[U_k, S_k, V_k] = \text{SVD}(K)$
$[U_m, S_m, V_m] = \text{SVD}(M)$
$L_f = S_{k,1,1} S_{m,1,1}$
$\hat{X}^{(k)} = 0$
$Z = 0$
$t = 1$
$i = 1$
while $i \leq i_{max}$ **do**
    $G = A^T(AZC - Y)C^T$
    $X_p \leftarrow \hat{X}^{(k)}$
    $\hat{X}^{(k)} = \text{prox}_{\text{SGL}}(\frac{\lambda}{L_f}, \lambda_2, \hat{X}^{(k)} - \frac{G}{L_f})$ (Algorithm S2)
    $t_p \leftarrow t$
    $t \leftarrow \frac{1+\sqrt{1+4t^2}}{2}$
    $Z \leftarrow X^{(k)} + \frac{t}{t_p}(X^{(k)} - X_p)$
    $i \leftarrow i + 1$
end while

Algorithm S2 Proximal of Sparse Group Lasso

Input: α, β, X
Output: $\text{prox}_{\text{SGL}}(\alpha, \beta, X)$

$P = \max(|X| - \alpha\beta)\text{sgn}(X)$
for rows P_i of P **do**
    if $\|P_i\| \geq (1-\alpha)\beta)$ **then**
        $P_i = \frac{1-(1-\alpha)\beta}{\|P_i\|} P_i$
    end if
end for
$\text{prox}_{\text{SGL}}(\alpha, \beta, X) = P$

S2 MERlin

Algorithm S3 MERlin decoding

Input: Y, C, bandpass filter h_b, PSF estimate h_p, decoding threshold t_d, min. cluster size c
Output: $\hat{X}$

$\hat{X} = 0_{N_h,f}$
for columns Y_i of Y **do**
  $Y_{f,i,:} = \text{vec}(\text{mat}(Y_i) * h_b)$
  $Y_{f,i,:} = \text{RL}(Y_{f,i,:}, h_p)$ (Richardson-Lucy deconvolution (Richardson 1972))
end for
for rows Y_j of Y **do**
  $d = \min_i \|C_{i,:} - Y_j\|^2$
  $\hat{i} = \arg\min_i \|C_{i,:} - Y_j\|^2$
  if $d < t_d$ **then**
    $\hat{X}_{j,i} = 1$
  end if
end for
for columns $\hat{X}_i$ of $\hat{X}^{(1)}$ **do**
  $B =$ `bwconncomp`$(\text{mat}(\hat{X}_i))$
  for clusters B_j in B **do**
    if $\text{size}(B_j) \geq c$ **then**
      $\mu_j = \text{round}(\text{centroid}(B_j))$
      $\hat{X}_{\mu_j,i} = 1$
    end if
  end for
end for

S3 Adaptive Filtering

To control for false positives, within each codebook several barcodes exist which are not assigned to any of the targeted genes. We bin the spots by the average value of the elements of $\boldsymbol{X}$ corresponding to the cluster, the cluster size and the average norm of the pixel traces, and for each bin compute the percentage of called spots which are assigned to blank barcodes. Then, the secant method is used to find a threshold on the acceptable percentage of blank barcodes which, when applied to the histogram, a specific misidentification rate of 0.05. Spots belonging to bins with a fraction of blank barcodes below this threshold are dropped from further analysis. Given a codebook with N_b blank barcodes and N_c coding (i.e. not blank) barcodes, and a dataset with B spots assigned to

blank barcodes and C spots assigned to coding barcodes, misidentification rate is defined as:

$$m = \frac{\frac{B}{N_b}}{\frac{C}{N_c}}. \tag{1}$$

.

S4 JSIT and BarDensr on simulated data

	Dataset 1			Dataset 2		
Pipeline	Precision	Recall	F-index	Precision	Recall	F-index
BarDensr	0.878	0.728	0.796	0.754	0.731	0.742
JSIT	0.932	0.779	0.848	0.952	0.746	0.837

S5 Supplemental Figures

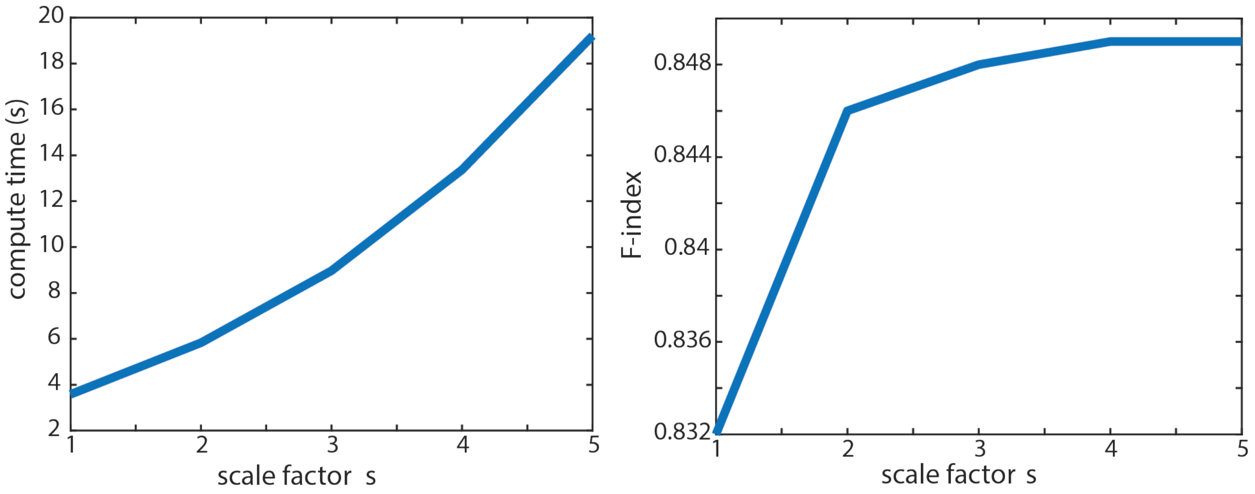

Figure S1: **Compute - performance trade off with scale factor** As the scale factor s increases, compute time increases (left). However, performance improves as s increases, as measured by F-index in decoding simulated data generated by the Bardensr package (Chen et al. 2021).

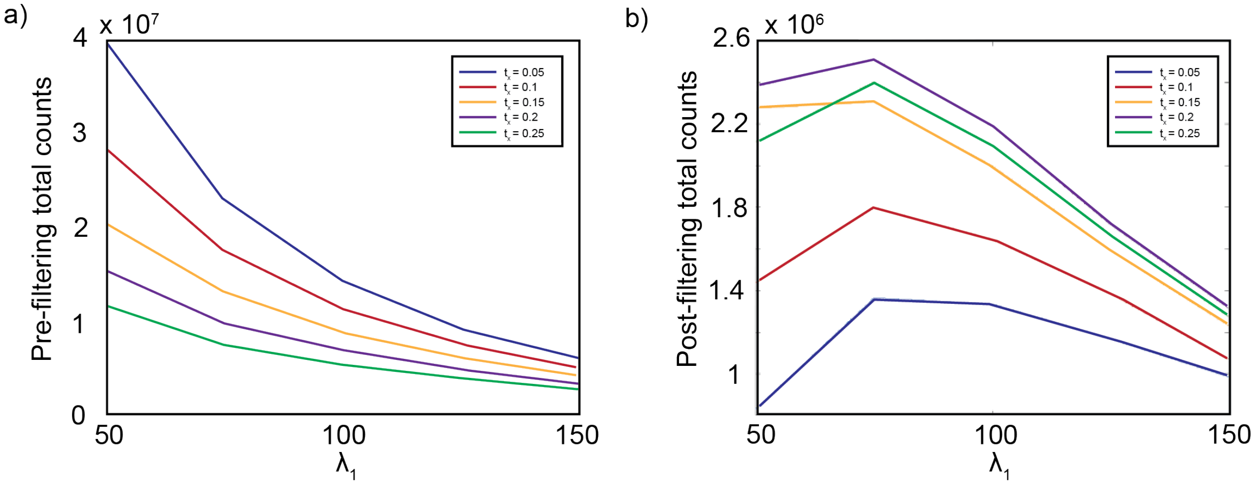

Figure S2: **Parameter selection of λ_1 and t_x** a) As λ_1 increases, total counts before adaptive filtering decreases. As t_x increases, total counts decrease. b) Below a certain point, decreasing λ_1 causes total counts after adaptive filtering to decrease. The same is true of t_x.

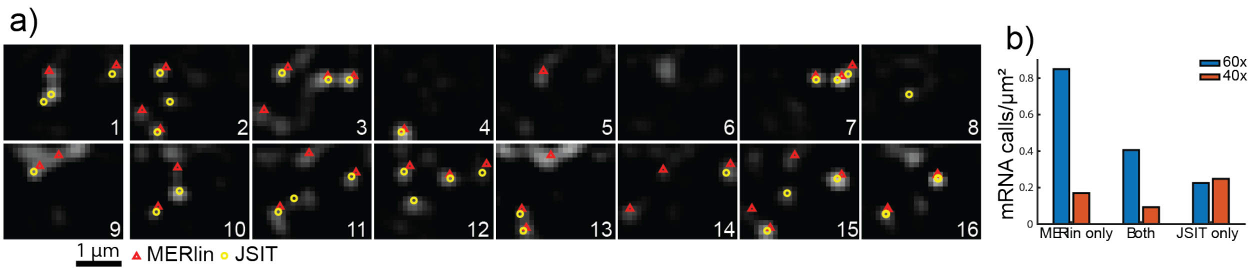

Figure S3: a) Imaging data from each of 16 frames of 60x MERFISH data, overlaid with estimates by MERlin and JSIT of positions of transcripts. b) Bar plot showing the number of transcripts per μm^2 identified by only MERlin, only JSIT, and by both pipelines, at both 60x and 40x.

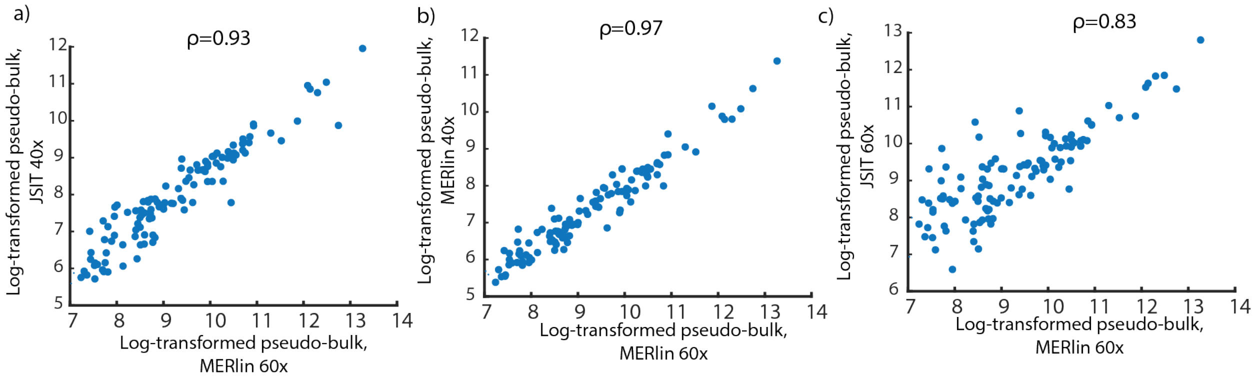

Figure S4: **Log-transformed pseudo-bulk expression by pipeline in MOp data** ρ is the Pearson correlation. Log-transformed pseudo-bulk gene expression between: a) MERlin 60x and JSIT 40x b) MERlin 60x and MERlin 40x c) MERlin 60x and JSIT 60x

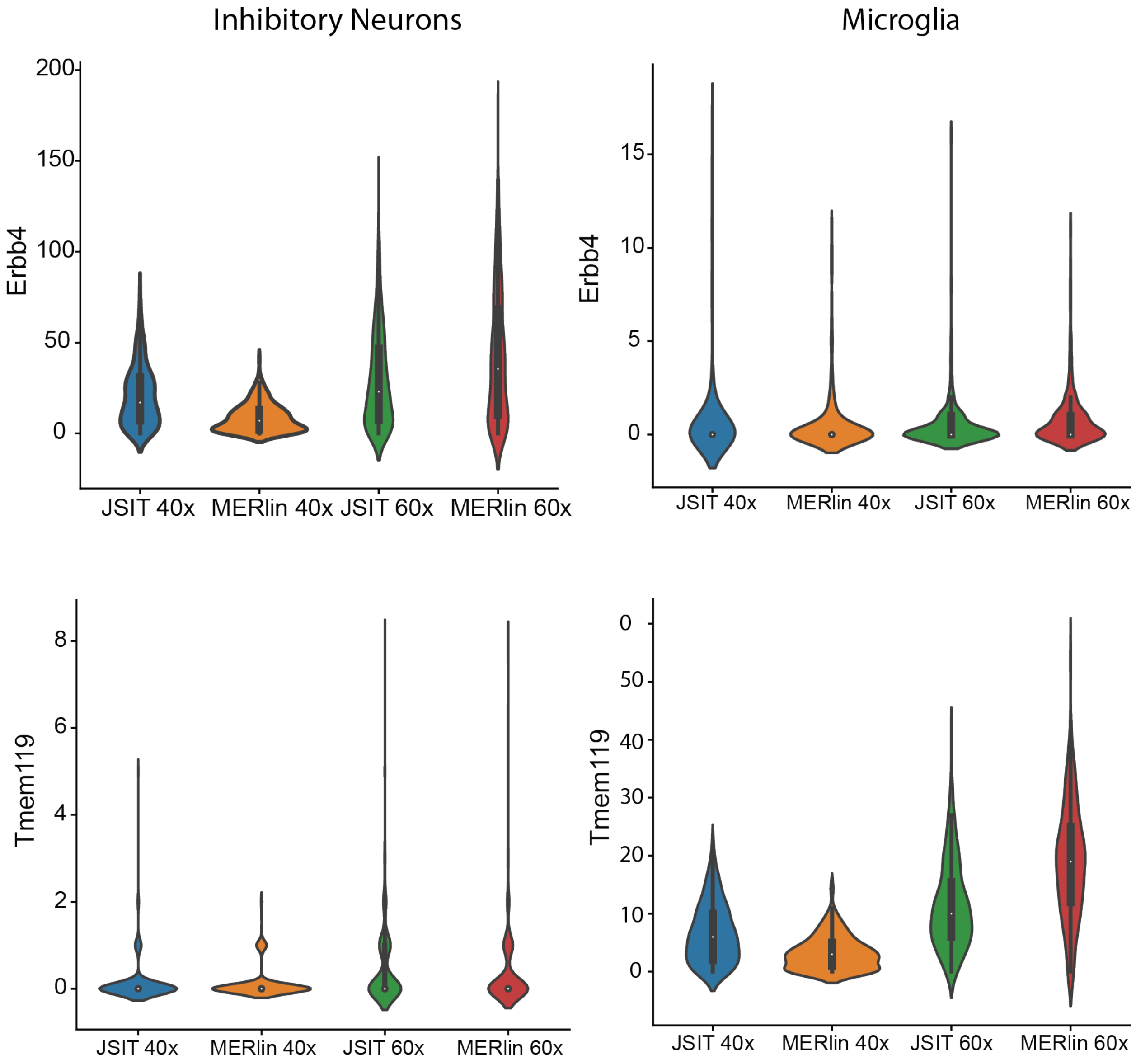

Figure S5: **Abundance of marker genes in different cell types** Violin plots showing expression of the genes *Erbb4*, a marker of inhibitory neurons, and *Tmem119*, a marker of microglia, in cells classified as inhibitory neurons and microglia. In all pipelines and at all magnification levels, expression of the marker genes is much higher in the corresponding cell type. Median calls of *Erbb4* in inhibitory neurons: JSIT 40x, 20.3, MERlin 40x, 8.5, JSIT 60x, 29.5, MERlin 60x, 41.6. *Erbb4* in microglia: JSIT 40x, 0.9, MERlin 40x, 0.5, JSIT 60x, 0.7, MERlin 60x, 0.8. *Tmem119* in microglia: JSIT 40x, 6.6, MERlin 40x, 3.2, JSIT 60x, 11.3, MERlin 60x, 18.8. *Tmem119* in inhibitory neurons: JSIT 40x, 0.1, MERlin 40x, 0.1, JSIT 60x, 0.5, MERlin 60x, 0.6.

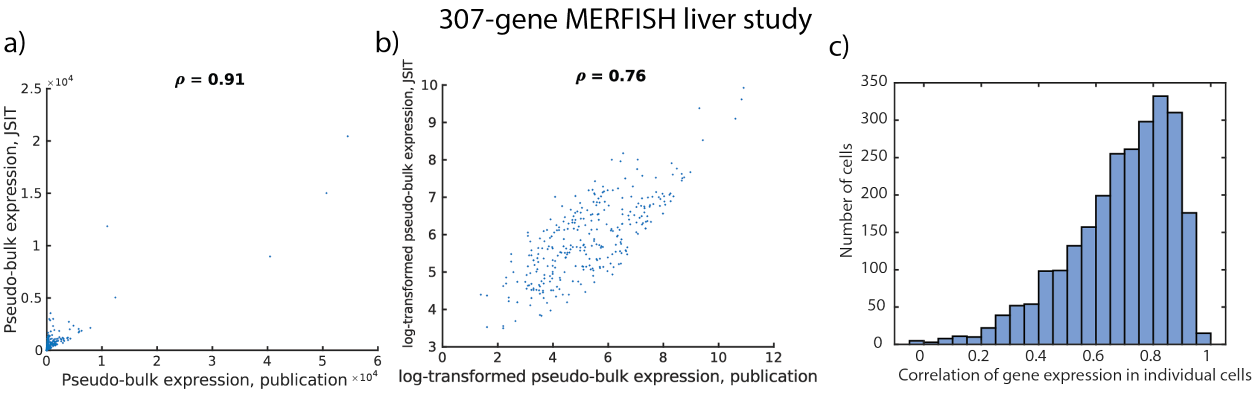

Figure S6: **JSIT decodes high-plex mouse liver MERFISH data** a) Pseudo-bulk gene expression comparison between JSIT and published data in 307-gene MERFISH mouse liver study. b) Log-transformed pseudo-bulk gene expression comparison. c) Histogram of gene expression correlation between JSIT and published data in individual cells.

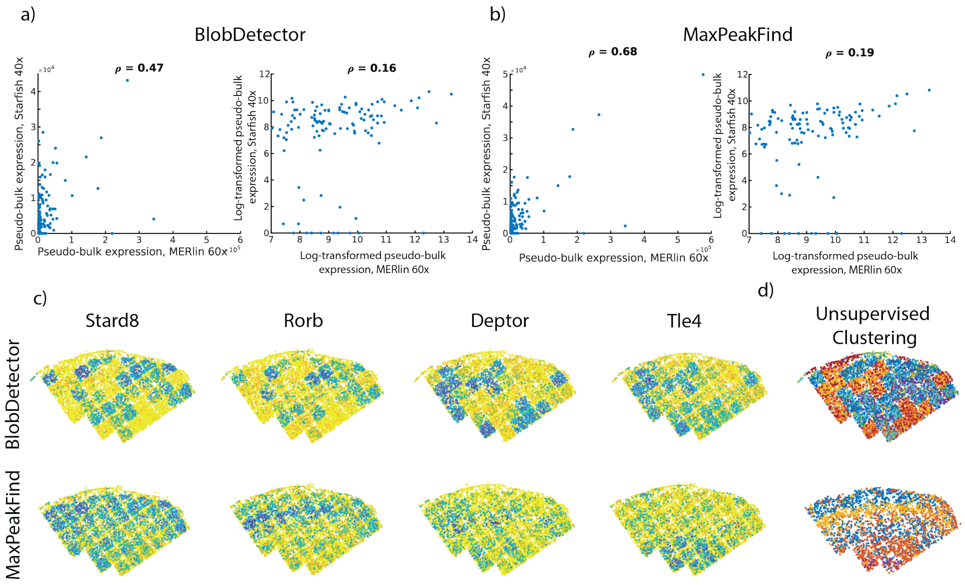

Figure S7: **Performance of Starfish decoding pipelines** a) Correlation of pseudo-bulk gene expression between BlobDetector results and MERlin 60x results. b) Correlation of pseudo-bulk gene expression between MaxPeakFind results and MERlin 60x results. c) Top: spatial expression patterns of marker genes of neuronal subtypes in 40x data decoded by the BlobDetector pipeline of Starfish, strong variation by FOV is exhibited. Bottom: spatial expression patterns of marker genes of neuronal subtypes in 40x data decoded by the MaxPeakFind pipeline of Starfish d) Top: Results of unsupervised clustering, BlobDetector. Bottom: results of unsupervised clustering, MaxPeakFind

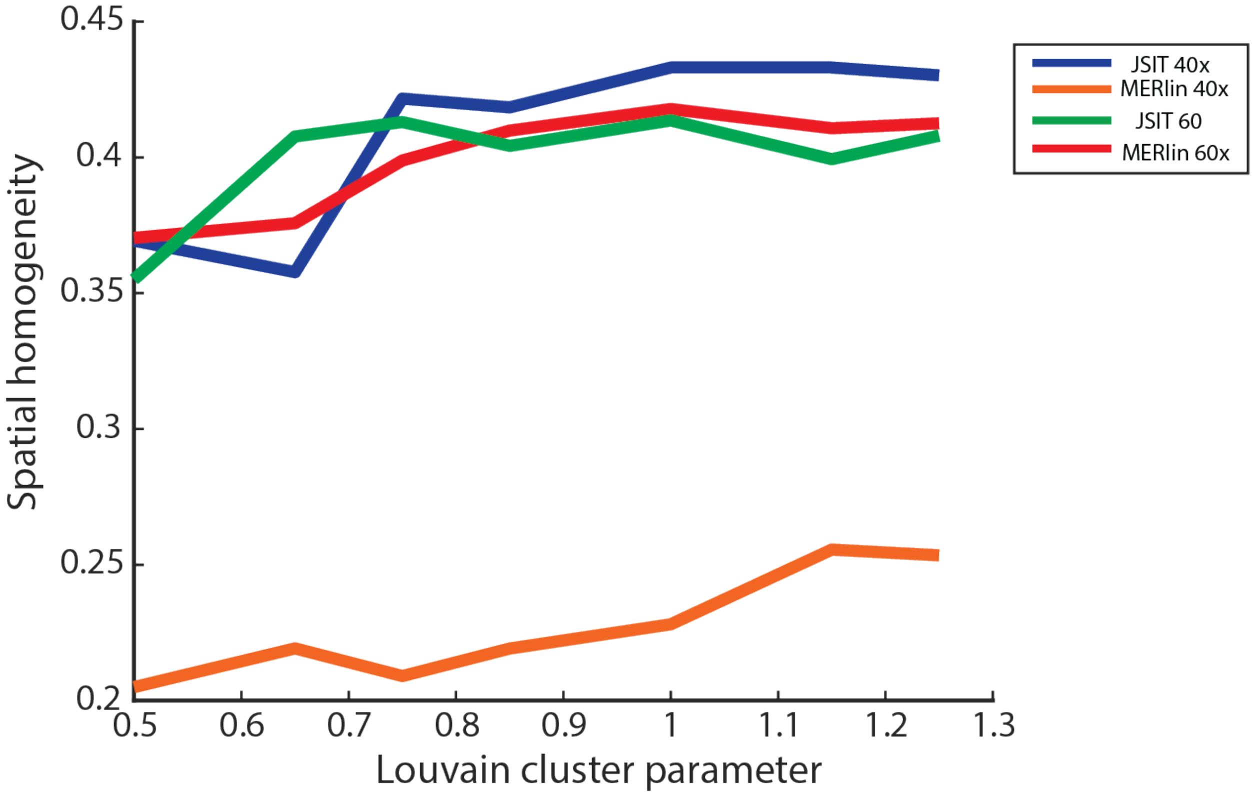

Figure S8: **Spatial Homogeneity varies with Louvain resolution parameter** As the Louvain resolution parameter increases, and clusters become more refined, spatial homogeneity tends to increase as well. However, the relative performance of the different pipelines and magnification levels remains quite consistent.

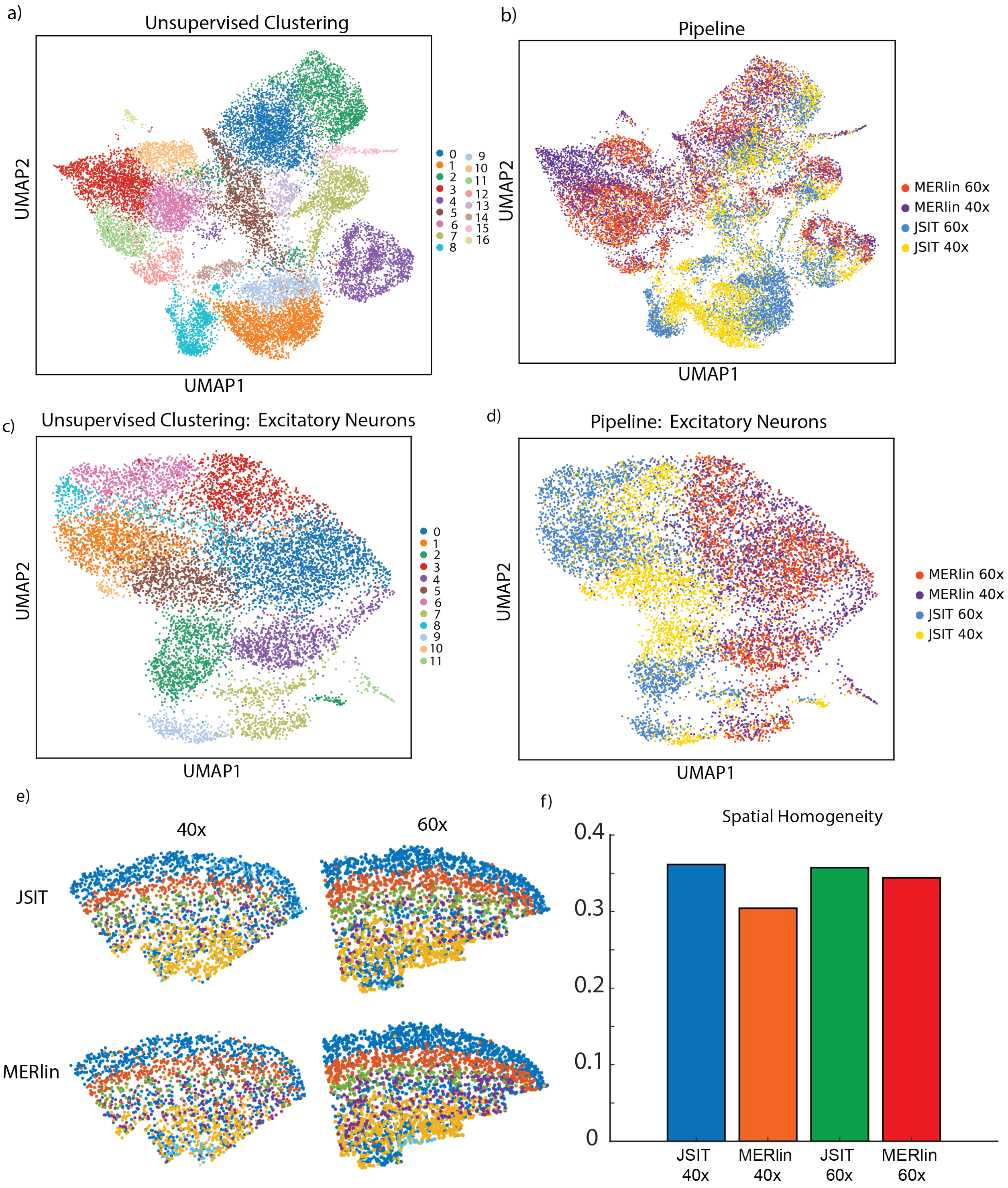

Figure S9: **Clustering JSIT and MERlin datasets together** a) Cell type clusters generated by Louvain clustering results from MERlin 60x, MERlin 40x, JSIT 60x, and JSIT 40x data together. b) Cells labeled by pipeline and magnification level. In many clusters, the four datasets are well-integrated. In clusters corresponding to excitatory neurons, MERlin 60x and 40x are clustered together, and JSIT 60x and 40x are clustered together. c) Subtypes generated by sub-clustering excitatory neuron clusters from the fours datasets together. d) Excitatory neurons labeled by pipeline and magnification level. JSIT results cluster together, and MERlin results cluster together. e) Spatial distribution of excitatory neuron subtypes. f) Spatial homogeneity scores for excitatory neuron subtypes, when clustered together.

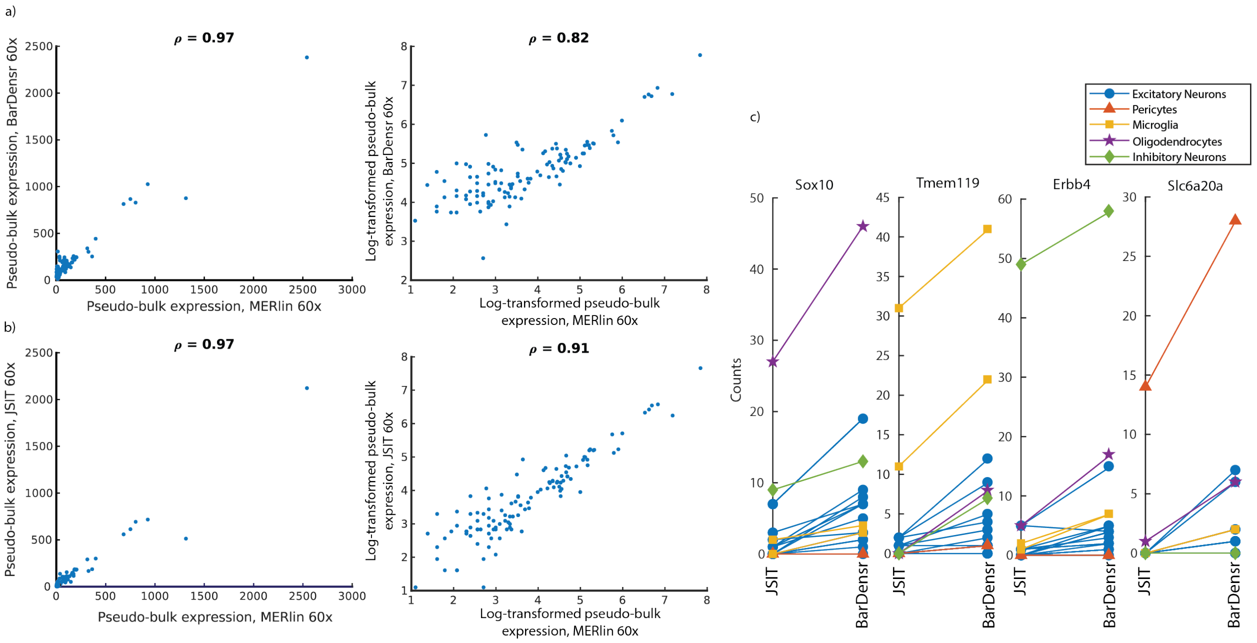

Figure S10: **Comparison of JSIT and BarDensr** a) Pseudo-bulk gene expression comparison between results of processing 4 1000x1000 pixel patches of mouse MOp MERFISH 60x data with BarDensr vs. MERlin. b) Pseudo-bulk gene expression comparison between processing the 4 patches with JSIT vs. MERlin. JSIT captures the expression of lower-expression genes more accurately than BarDensr. c) Expression of marker genes *Sox10* (oligodendrocytes), *Erbb4* (inhibitory neurons), *Tmem119* (microglia), and *Slc6a20a* (pericytes) in 19 cells across the 4 patches in both BarDensr and JSIT results. BarDensr exhibits higher counts of the marker genes in cells which do not appear to correspond to the cell type.

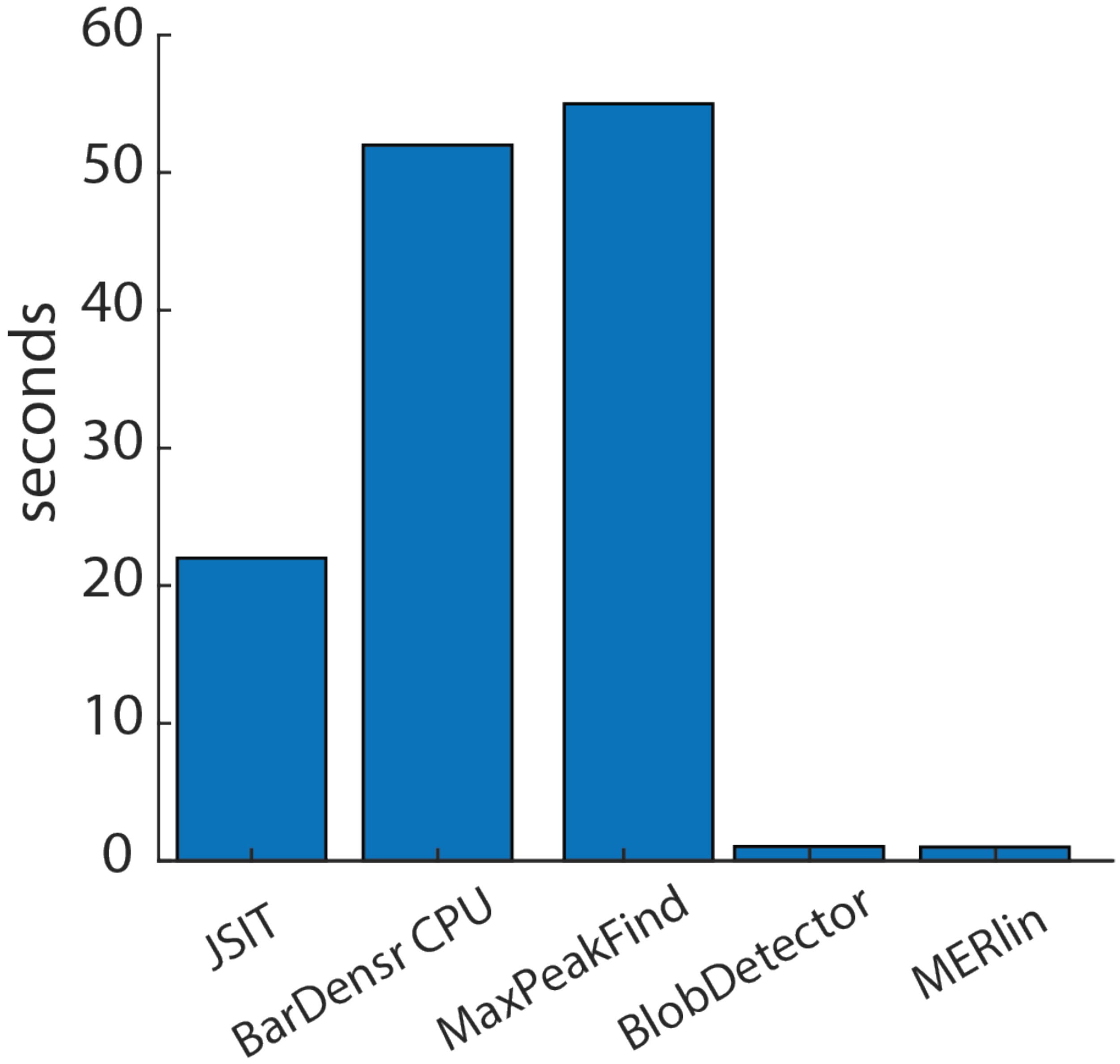

Figure S11: **Compute time requirements of decoding pipelines** Compute time required to decode a 200x200 pixel patch of iST data, in the decoding pipelines tested.

References

Chen, S. et al. (2021). "Barcode demixing through non-negative spatial regression (bardensr)". In: *PLoS computational biology* 17.3, e1008256.

Richardson, W.H. (1972). "Bayesian-Based Iterative Method of Image Restoration". In: *J. of Optical Soc. of America* 62, pp. 55–59.
